# Rheumatism and Its Treatment

**Published:** 1907-05

**Authors:** 


### RHEUMATISM AND ITS TREATMENT.

F. J. Walter declares that the various infections will account for every form of so-called "rheumatism," except muscular, and that is an intoxication. This intoxication accompanies or precedes most articular, and some nerve infections. The term "rheumatism" is a misnomer. Intelligent treatment consists of prophylaxis, a better understanding between patient and physician, with attention to social conditions, dietetics, exercise, or rest, as indicated, elimination by proper baths, fresh air, the right co-operative mental attitude, and in some cases climate. An examination of the urine is important in every case. Heredity has no effect, except as establishing social conditions followed by the family. A sedentary life, and also great muscular fatigue, should be avoided, the latter being a cause of muscle pains in children and working men. Alkaline waters and drugs, although greatly abused, are very important antacids and antiseptic to the intestines.—Medical Record, January 19, 1907.

